# Social Learning in the Ultimatum Game

**DOI:** 10.1371/journal.pone.0074540

**Published:** 2013-09-04

**Authors:** Boyu Zhang

**Affiliations:** School of Mathematical Sciences, Beijing Normal University, Beijing, P.R. China; University of Maribor, Slovenia

## Abstract

In the ultimatum game, two players divide a sum of money. The proposer suggests how to split and the responder can accept or reject. If the suggestion is rejected, both players get nothing. The rational solution is that the responder accepts even the smallest offer but humans prefer fair share. In this paper, we study the ultimatum game by a learning-mutation process based on quantal response equilibrium, where players are assumed boundedly rational and make mistakes when estimating the payoffs of strategies. Social learning is never stabilized at the fair outcome or the rational outcome, but leads to oscillations from offering 40 percent to 50 percent. To be precise, there is a clear tendency to increase the mean offer if it is lower than 40 percent, but will decrease when it reaches the fair offer. If mutations occur rarely, fair behavior is favored in the limit of local mutation. If mutation rate is sufficiently high, fairness can evolve for both local mutation and global mutation.

## Introduction

Ultimatum game introduced by Guth et al. [1] is one of the most influential games in experimental economics that people in the real world do not behave rational. The setting of the game is quiet simple. Two players divide a sum of money. The proposer makes an offer on how to split and the responder decides whether to accept. If the offer is rejected, both players get nothing. A rational responder ought to accept any non-zero offer. Therefore, a selfish proposer who thinks that the responder is rational should offer the minimal. Game theory predicts the rational outcome, however, empirical studies in human society, including both laboratory games and field games, prefer fair outcome. In hundreds of ultimatum games conducted in different countries in the last 30 years, proposers on average offer 40 to 50 percent of the total sum to the responder. Responders usually accept offers higher than 40 percent and about half of all responders reject offers below 30 percent [2]–[7].

How to understand people rejecting positive offers? One well known economic explanation is that irrational individuals have preference on fairness [8]–[9]. In these models, utility functions of players depend not only on their own payoff but also the payoff of the others. Responders reject low offers because the disutility of receiving a payoff less than the proposer is greater than the utility of getting small monetary benefits. On the other hand, the rejection of a unfair offer can be seen as a kind of punishment that inhibits selfish behaviors in later rounds. In iterated ultimatum game experiments, average offers are much more closer to the fair share [9]–[12]. However, this contradicts the equilibrium analysis since the only subgame perfection is not to reject.

From the perspective of evolutionary game theory, replicator dynamics, which assumes that successful strategies will always come to dominate the population, favors low offers and demands [13]–[16]. Therefore, to explain fairness using evolutionary dynamics, additional mechanisms are necessary. One approach involves reputation system. If proposers can obtain information about responders’ demands and believe that responders reject offers lower than their aspiration level, high offers and demands will prevail the population [14], [16]. Spatial structure was also found to play a key role in the evolution of fairness. Pioneering work by Page et al. [17] pointed out that ultimatum game on ring and lattice may evolve to much fairer outcomes compared with random encounter setting. Subsequent research confirmed that fairness is enhanced in heterogenous graphs [18]. Furthermore, spatial ultimatum game with discrete strategies exhibits fascinatingly rich dynamical behavior such as traveling waves and cyclic dominance [19], and the fair solution is obtained in the limit of a continuous strategy set [20]. A third approach emphasizes the importance of randomness [13], [21]. By using stochastic evolutionary game theory, Rand et al. [21] demonstrated that natural selection favors fair behavior when sufficient randomness is present.

In this paper, we study the iterated ultimatum game by a learning-mutation process. To analyze the game, define individual strategy as 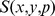, meaning giving 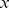 of the total sum to the responder when acting as a proposer and rejecting any offer less than 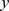 with probability 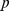 (and accepting offers equal or higher than 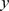 with probability 1) when acting as a responder, where 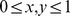 and 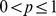. The parameter 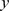 can be seen as proposer’s demand or aspiration level. Following this definition, the rational strategy is written as 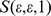, where 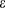 is the minimum offer greater than 0, and the fair strategy is 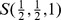. In our model, individuals update their strategies through quantal response learning. We will show that learning-mutation process leads to oscillations from offering 0.4 to 0.5 and rejecting lower offers with probability one half.

## Methods

Before study the ultimatum game with continuous strategies, we first consider the iterated mini ultimatum game with only two possible offers 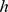 and 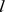, with 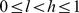 [13]–[14], [16], [22]–[23]. In each round, the proposer has to choose the high offer 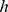 (labeled by 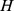) or the low offer 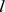 (labeled by 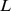), and the responder has to decide to reject the low offer 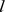 (labeled by 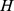) or accept (labeled by 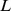). The payoff matrix is then written as
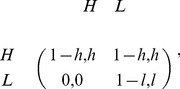
(1)where the proposer plays rows and the responder plays columns. The mini game has a strict Nash equilibrium 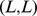, and non-isolated Nash equilibria 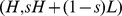, where 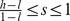. Notice that each equilibrium 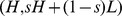 is weakly dominated by 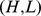, 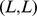 is the only subgame perfection. Therefore, rational players will choose 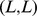 according to backward induction.

Payoff matrix (1) can also be interpreted as the Prisoner’s Dilemma game with punishment, where higher offer and low offer correspond to cooperation and defection, respectively, and rejecting the low offer means paying 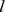 to punish defector 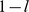 [16], [22]. Similarly as the mini ultimatum game, 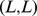 is the only subgame perfection.

There are many ways to model social learning [10], [13], [24]–[26]. In this paper, we apply the well-known quantal response equilibrium (QRE) introduced by McKelvey and Palfrey [27]–[29]. In a QRE, players are assumed boundedly rational and do not always choose best responses. Instead, they make decisions based on probabilistic choice functions and believe other players do so as well. A general interpretation of this model is that players observe random perturbations on the payoffs of strategies and choose optimally according to those noisy observations. The most common specification of QRE is the logit equilibrium, where noises follow the extreme value distribution [30]–[32]. Let 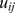 denotes the expected payoff of player 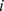 using strategy 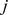 (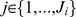). The logistic response function is defined as
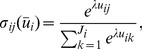
(2)where 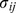 is the probability that player 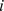 adopts strategy 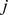 and 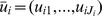. If each player uses a logistic response function, QRE or logit equilibria are the solutions of 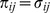, where 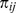 is the frequency of strategy 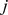 in player 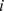. The logistic response function has one free parameter 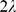, who has been interpreted as the intensity of selection [16], [21]. At 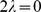, players have no information about the game and each strategy is chosen with equal probability. As 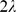 approaches infinity, players achieve full information about the game and play the best response.

The quantal response method has been widely used to explain experimental data. For instance, Yi [29] applied QRE to fit data of high stakes ultimatum games [33]. In iterated games, estimates of 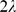 usually increase as the game progresses [27]–[29]. As players gain experience from repeated observations, they can be expected to make more precise estimates and finally reach a Nash equilibrium. To describe this process, consider QRE as a function of 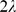. When 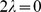, the QRE is at the centroid of the strategy simplex, and when 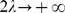, the QRE set consists of Nash equilibria only. As pointed out by McKelvey and Palfrey [27], for almost all normal form games, the graph of logit equilibria correspondence contains a unique branch which starts for 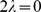 at the centroid and converges to a unique Nash equilibrium as 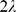 goes to infinity. This then defines a unique selection from the set of Nash equilibria by “tracing” the graph of the logit equilibrium correspondence starting at the centroid. The selected Nash equilibrium is called the *limiting logit equilibrium* (LLE) of the game.

## Results

For the mini ultimatum game Eq.(1), logit equilibria are solutions of
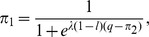


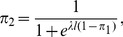
(3)where 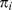 denotes the probability of player 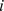 using his first strategy and 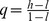. When 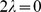, Eq.(3) has a unique solution 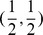, and when 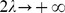, the QRE set consists of three Nash equilibria only, (0,0) 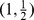 and 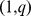 (see Figures S1 and S2). In general, the LLE is one of two Nash equilibria, either 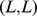, giving the low offer and accepting the low offer, or 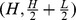, giving the high offer and rejecting the low offer with probability one half. Approximately, the LLE is 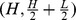 if and only if



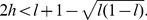
(4)(See Text S1).

**Figure 1:**
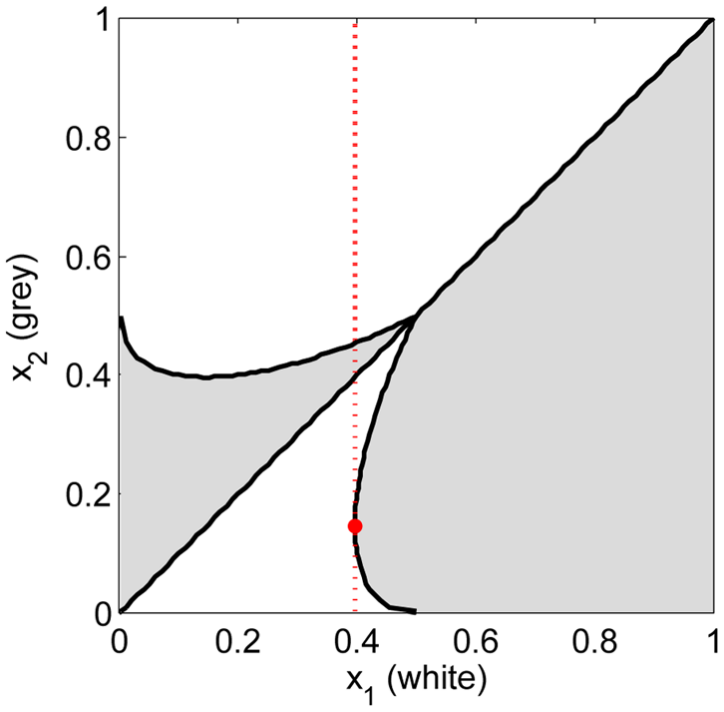
Pairwise invasibility plot. 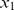
 and 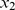 are dominant in white and gray regions, respectively. The red dash line denotes 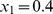 and the red point denotes 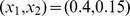. Every offer 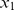 lower than 0.5 is dominated by some higher offers and 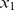 equal or greater than 0.4 is also dominated by lower offers. In particular, 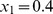 dominates almost all lower offers, the only exception is 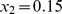 (the red point).

If the high offer is the fair offer, i.e., 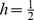, Eq.(4) tells us that social learning chooses the low offer. In fact, any high offer equal or greater than 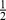 is unfavored. On the other hand, if the low offer is the rational decision, i.e., 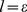, any high offer smaller than 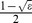 is selected. Therefore, social learning does not always prefer the rational outcome. For convenience, we say that offer 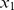 *dominates* offer 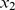 if 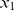 is the LLE of the mini ultimatum game with two offers 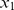 and 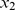. Dominant regions of 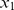 and 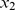 are shown in Figure 1. Offers lower than 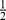 are dominated by slightly higher offers. For 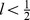, the right side of Eq.(4) is a convex function, where at the minimum 
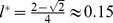
 and 
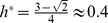
. This implies that if 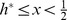, 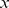 is also dominated by some low offers. In particular, 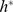 dominates almost all lower offers (the only exception is 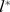, see the red point in Figure 1).

**Figure 2:**
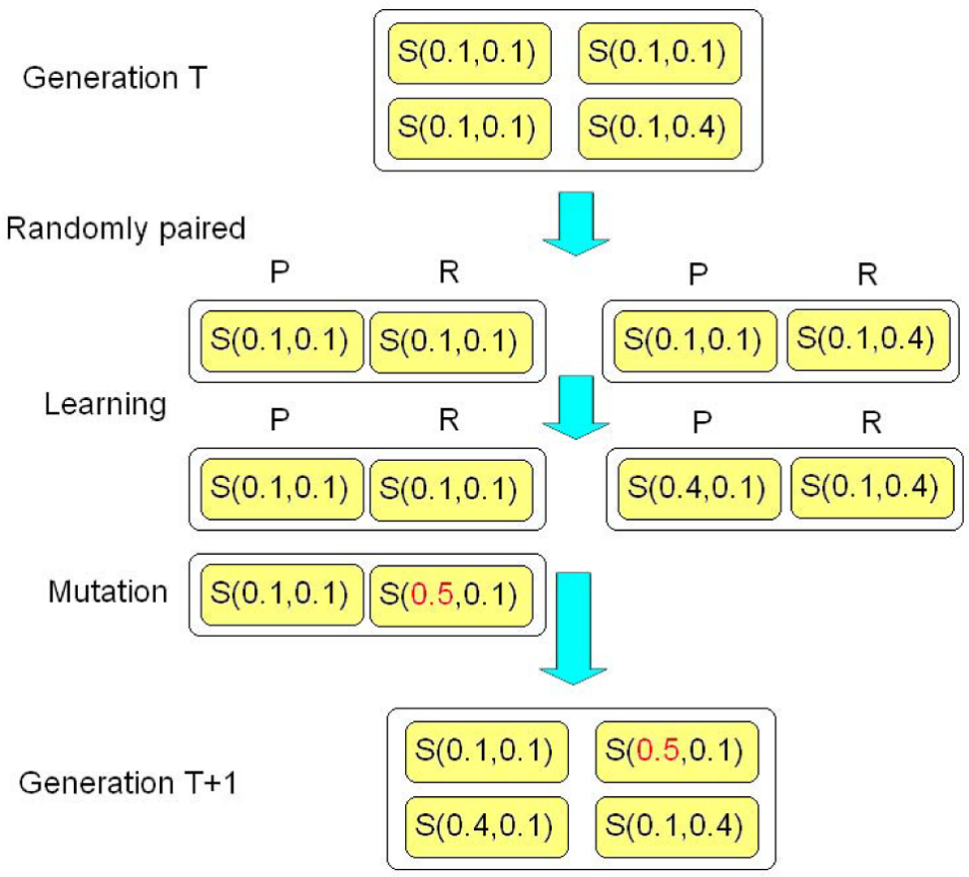
An example for the learning-mutation process. An example for the learning-mutation process in a population of four players from generation 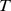 to generation 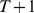. In generation 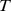, three players adopt 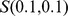 and one adopts a mutant strategy 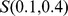. At the beginning, they are divided into two mini ultimatum games, 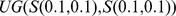 and 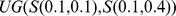, and update their strategies by the quantal response learning (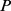 means proposers and 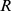 means responders). In the first group, players do not change their original strategies, while in the second group, the proposer will change his strategy to 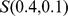 since 0.4 dominates 0.1. Mutations happen after all the pairs reach Nash equilibria. The responder in the first pair mutates to 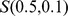 (the red number). As a result of learning and mutation, strategies in generation 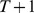 are 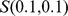, 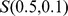, 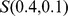 and 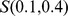.

Let us now introduce the learning-mutation process on the continuum of all strategies. Consider a population of 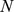 players. In each generation, players are randomly paired and play the iterated mini ultimatum game. We begin by considering the single role mode, in which roles of two members in a group are decided randomly before the game starts and do not change in an interaction, and discuss later that main results are qualitatively unchanged if they play both roles. Two players update their strategies by the quantal response learning and the iterated game will stop if they reach a Nash equilibrium since in this situation both are unwilling to change. Mutations happen after all the groups reach Nash equilibria. With probability 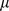, players adopt a new strategy. In local mutation scenario, they plus or minus a small random value on their former strategies, and in global mutation scenario, all strategies are drawn with equal probability (See Figure 2).

We first look at the learning process in one generation. Denote the mini ultimatum game where the proposer using strategy 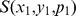 and the responder using strategy 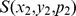 by 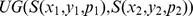. In this game, the proposer offers 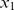 and the responder rejects offer lower than 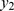 with probability 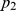. The payoff of this game is described by (1), where 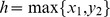 and 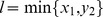. At the beginning, both players have the motivation to adjust their strategies. If 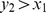, 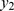 is the high offer 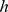 and 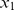 is the low offer 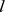. The proposer tends to increase his offer from 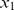 to 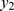 in order to avoid being refused, and meanwhile, the responder tends to decrease his demand from 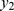 to 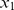. Conversely, if 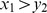, the proposer wants to decrease his offer from 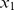 to 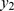 and the responder wants to increase his demand from 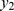
z to 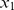.

According to the quantal response learning, both players choose their initial action randomly, i.e., the proposer suggests the lower offer with probability one half and the responder rejects this offer with the same probability. An approximated formula to decide the LLE is provided by Eq.(4). At the LLE, the responder either accepts the low offer or rejects the low offer with probability one half. This implies that the rejection rate 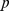 will converge to 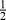 in the long run. For simplicity, we write 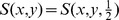 in later discussions. At the end of game 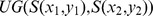, if 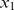 dominates 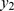, the proposer keeps his strategy unchanged but the responder adopts a new strategy 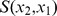. However, if 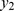 dominates 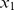, the responder’s strategy does not change but the proposer adopts a new strategy 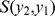. We observe that the diversity of offers decreases from one generation to another since in each mini game, one dominated offer is eliminated. In fact, for any (heterogenous) population, quantal response learning will eventually lead to a homogenous self-consistent population in which each player’s offer equals to his demand.

The same result arises if players act both roles. To see this, suppose that two players using strategies 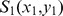 and 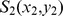 are randomly paired. In the interaction, they will play two games 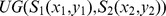 and 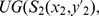 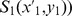, where player 1 acts as a proposer in the first game and acts as a responder in the second game, and 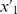 and 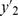 denote respectively the offer of player 1 and the demand of player 2 after the first game. Notice that 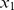 competes with 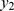 in the first game and 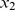 competes with 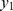 in the second game, the final outcome does not change with the order of two games, and two dominated offers are eliminated at the end of the interaction. Thus, similarly as the single role mode, the diversity of offers decreases and the population will converge to a homogeneous state.

**Figure 3:**
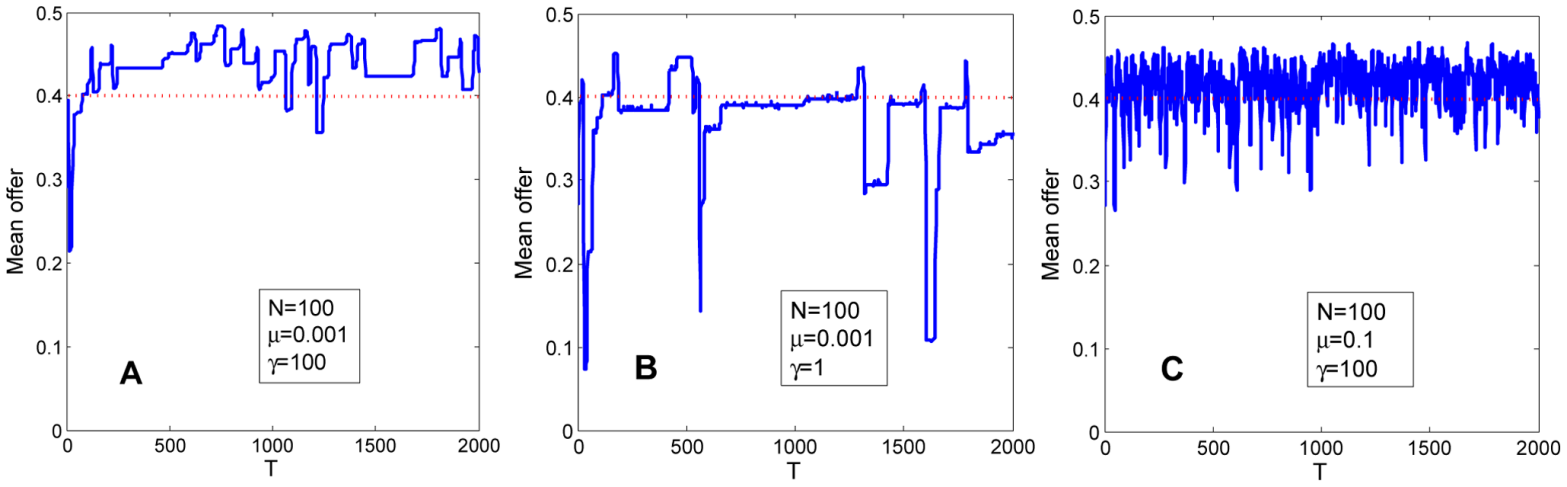
Time evolution of the population mean offer. The population size is 100 and evolves under the learning-mutation process. Mutation rates are taken as 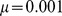 (weak mutation rate) in Figures 3A and 3B and 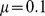 (intermediate mutation rate) in Figure 3C. Mutant strategies follow 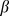-distributions, where 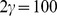 (local mutation) in Figures 3A and 3C, and 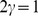 (global mutation) in Figure 3B. Red dash lines denote 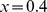. In all figures, the population mean offer increases if it is smaller than 0.4 but oscillates if it is between 0.4 to 0.5.

Let us now add the possibility of mutation. We first look at the limit of weak mutation rate 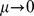. As in the adaptive dynamics model, mutations occur rarely so that a mutant will either vanish or has taken over the population before the next mutation happens [15], [34]–[35]. Under weak mutation, the population is homogeneous in most of time, and we represent the strategy of the residents 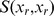 by 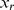. Eq.(4) indicates that (a) if 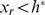, the population could only be replaced by mutants using offers higher than 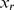, (b) if 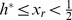, both higher and lower offers may invade, (c) if 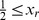, any lower offer could take over the population and higher offer can not invade (See Figure 1). Generally speaking, the learning-mutation process leads to oscillations in interval 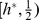, where proposers offer 40 to 50 percent of the total sum to responders and responders reject offers below their expectation with probability one half. Once the resident strategy leaves the interval, learning and mutation will push it back (see Figure 3).

**Figure 4:**
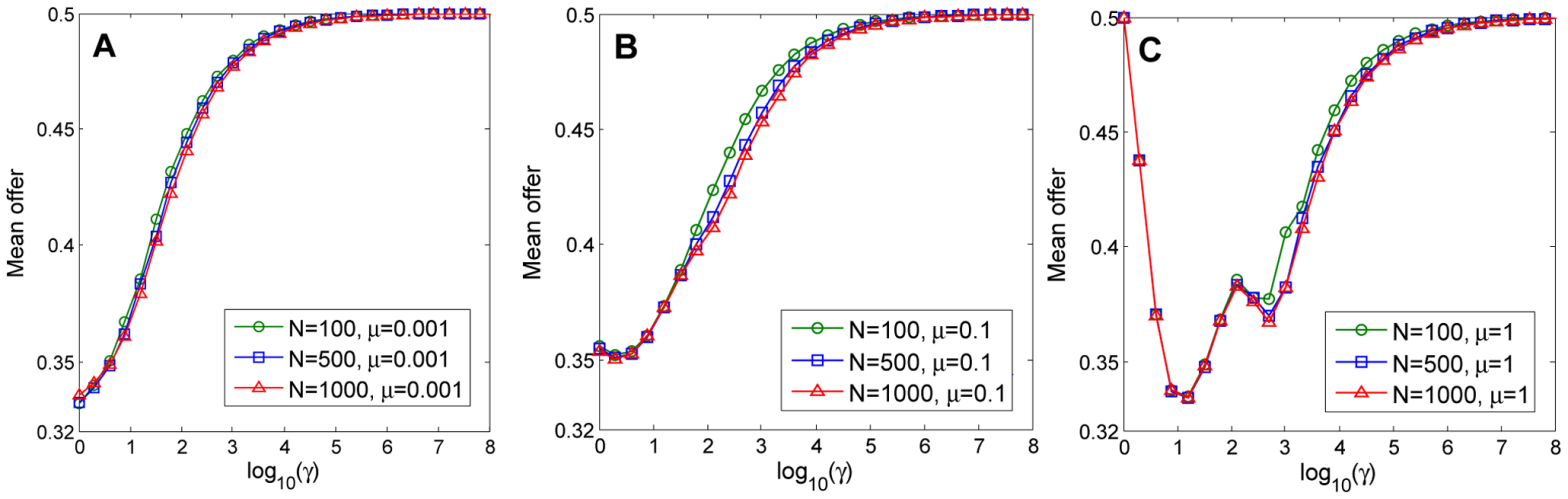
Effects of mutations and population sizes on the long-term mean offer. Mutation rates are taken as 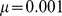 (weak mutation rate), 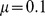 (intermediate mutation rate) and 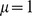 (high mutation rate) in Figures 3A, 3B and 3C, respectively. The long-term mean offer depends significantly on the mutation rate and the mutation range, but is robust to different population sizes.

The long-term mean offer of the population depends significantly on the range of mutation. In local mutation scenario where the mutational jumps are small such that the resident strategy changes continuously, the time evolution of 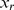 could be described by the adaptive dynamics [15], [34]–[35]. It is easy to verify that 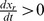 if 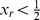 but 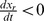 if 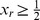. 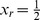 is a degenerate point of the adaptive dynamics, i.e., the resident strategy will decrease when it reaches the fair offer. In this case, the long-term mean offer is slightly lower than 0.5 (see Figure 4A). In contrast, if mutations are global that are picked from the uniform distribution [0,1], 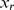 may drop dramatically when it is greater than 0.4 (see Figure 3B). Thus, broader range of mutation leads to lower mean offer. To illustrate this intuition, suppose that mutant strategies in 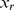 population are picked from a 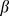-distribution Beta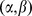, where 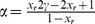 and 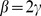 [21]. The particular values of 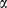 and 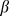 are chosen such that the modal value of the distribution is 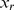. The inverse of 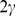 measures the range of mutation, where 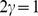 means uniform distribution on [0,1] and 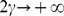 means local mutation. Figure 4A shows clearly that the long-term mean offer of the population is monotonically increasing in 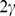. When 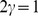, the mean offer is about 0.32, and when 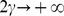, it converges to the fair outcome 0.5.

In the high mutation limit 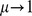 with global mutation 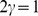, all strategies are present in the population simultaneously with approximately equal frequency and therefore the long-term mean offer is exactly 0.5 [21]. As 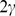 increase, the mean offer will first decline to below 0.4. This follows from the fact that a successful strategy should maximize the winning probability when playing against a randomly chosen strategy. As shown in Figure 1, offers around 0.4 have smaller dominated regions therefore are preferred in a random world. However, when the mutation range becomes narrow, the mean offer will increase since any offer lower than 0.5 are dominated by slightly higher offers. Finally, it converges to 0.5 as 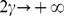 (see Figure 4C).

At intermediate mutation rates 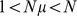 where mutations happen frequently and the population has a high diversity of strategies, the time evolution of the one generation population mean offer could be characterized by the theoretical predictions of the weak mutation limit [15]. That is, the one generation mean offer increases if it is smaller than 0.4 but oscillates if it is between 0.4 to 0.5 (see Figure 3C). Although a smaller population converges to homogeneous state easier than a larger population, numerical simulations suggest that the long-term mean offer is robust to different population sizes (see Figure 4). In particular, intermediate mutation rates result in intermediate mean offer. For global mutation 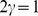, the long-term mean offer is higher than that of weak mutation limit 0.32 but lower than that of high mutation limit 0.5, and when 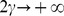, the long-term mean offer goes to 0.5 as the two extremes (see Figure 4B).

## Discussion

Similarly as [14], we assume that a proposer knows what offers have been accepted by the responder in the last generation. This information can be easily obtained since every group reaches an agreement on a certain offer at the end of a generation. Given this information, the proposer should either to stay unchanged or to adopt the responder’s strategy. In fact, switching to any other offer is unreasonable: giving offers lower than the demand will face the risk of being rejected and giving offers higher than the demand is inefficient. If the ultimatum game is anonymous such that the responder’s demand is unknown to the proposer, the proposer then chooses offers from interval [0,1]. In this case, the LLE of the ultimatum game is the rational equilibrium, where the proposer offers the minimal and the responder accepts any non-zero offer [29].

A difference between our model and [14] is that we include the possibility that responders accept lower offers. Consequently, proposers have to evaluate whether to satisfy responders’ demands. According to quantal response learning, two players in a group choose their initial strategies randomly. The motivation is twofold. On the one hand, each player faces a new game in a new interaction since the payoff matrix of the mini ultimatum game is decided by the strategies of both players. On the other hand, empirical evidences from the repeated Prisoner’s Dilemma games (with punishment) support this consideration. The frequency of cooperation (which corresponds to the frequency of high offer in the mini ultimatum game) in the initial round of each interaction is nearly the same and decreases over rounds [3], [25], [36]–[37]. We can then expect that players are affected little by past experience and update their strategies entirely by social learning.

We consider that players are boundedly rational and choose the best response according to noisy observations. In an interaction, two players update their strategies simultaneously. At the beginning, the proposer is inclined to make the high offer due to the high rejection rate and the responder tends to accept the lower offer since rejecting is costly. Observation errors decrease as the game progresses and two players will finally reach a Nash equilibrium. Intuitively, their strategies converge to the high offer if the proposer learns faster than the responder, i.e., the proposer stops making the low offer before the responder stops rejecting. This happens when the low offer is small, which means the rejection of the low offer causes a greater loss to the proposer than to the responder. Thus, mistakes in evaluating the payoffs of strategies lead to fairer outcome.

We note that there exist at least two theoretical papers investigated the effects of randomness on ultimatum game [13], [21]. Gale et al. [13] studied noisy replicator dynamics with asymmetric mutation structure in which responders attempt to adopt new strategies more often than proposers. The greater variation in responder behavior then forces proposers to make higher offers. Rand et al. [21] analyzed the stochastic evolutionary dynamics under weak selection. They showed that larger mutation rate could lead to a heterogeneous population with higher average offer and acceptance level. In the high mutation limit, as has been shown in [17], the prevailing strategy is 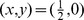, while in the low mutation limit, the prevailing strategy is 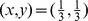. Furthermore, their results are robust to the range of mutation.

In our paper, the intensity of selection is weak at the beginning of an interaction and increases as players gain experience from repeated observations. At the end of the interaction, the selection is strong and players only choose the strategies with the highest payoff. Different from [21], the long-term mean offer is affected by both the mutation rate and the mutation range. For low and intermediate mutation rates, the mean offer increases with decreasing mutation range, and fair behavior is favored in the limit of local mutation. In contrast, for high mutation rates, both global mutation and local mutation lead to fair outcome.

The emergence of equity is as complicated as the evolution of human society. Our model excluded many important issues, such as preference on fairness [8]–[9] or punishment [38]–[40], social networks [17]–[20], emotions [41]–[42] and culture difference [4]–[6], [43]. Based on learning and mutation, we show that individuals entirely motivated by self interests can evolve toward fairness.

## Supporting Information

Figure S1
**Graph of QRE correspondence.** Parameters are taken as 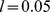, 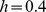 in Figure S1A and 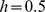 in Figure S1B. Blue curve and red curve are 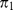 and 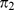, respectively. In Figure S1A, since 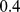 dominates 0.05, 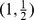 is the LLE. In Figure S1B, since 0.5 is dominated by 0.05, (0,0) is the LLE.(TIF)

Figure S2
**Graph of Eq.(S7).** Parameters are taken as 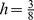 and 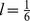, i.e., 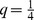. 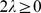 on solid curves but 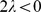 on the dashed curve. Black points are Nash equilibria and the red point is the LLE. The graph of Eq.(S7) consists of two branches, where one passes through the Nash equilibrium 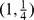 and the other passes through the centroid 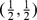. Since 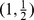 and 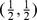 are on the same branch, higher offer is the LLE.(TIF)

Text S1 **Supporting Information for Social learning in the ultimatum game.**
(PDF)
