# Supplementary figures and images for: Social Learning in the Ultimatum Game
Source: PLoS One. 2013 Sep 4;8(9):e74540. doi: 10.1371/journal.pone.0074540 (PMC3762740; doi:10.1371/journal.pone.0074540)

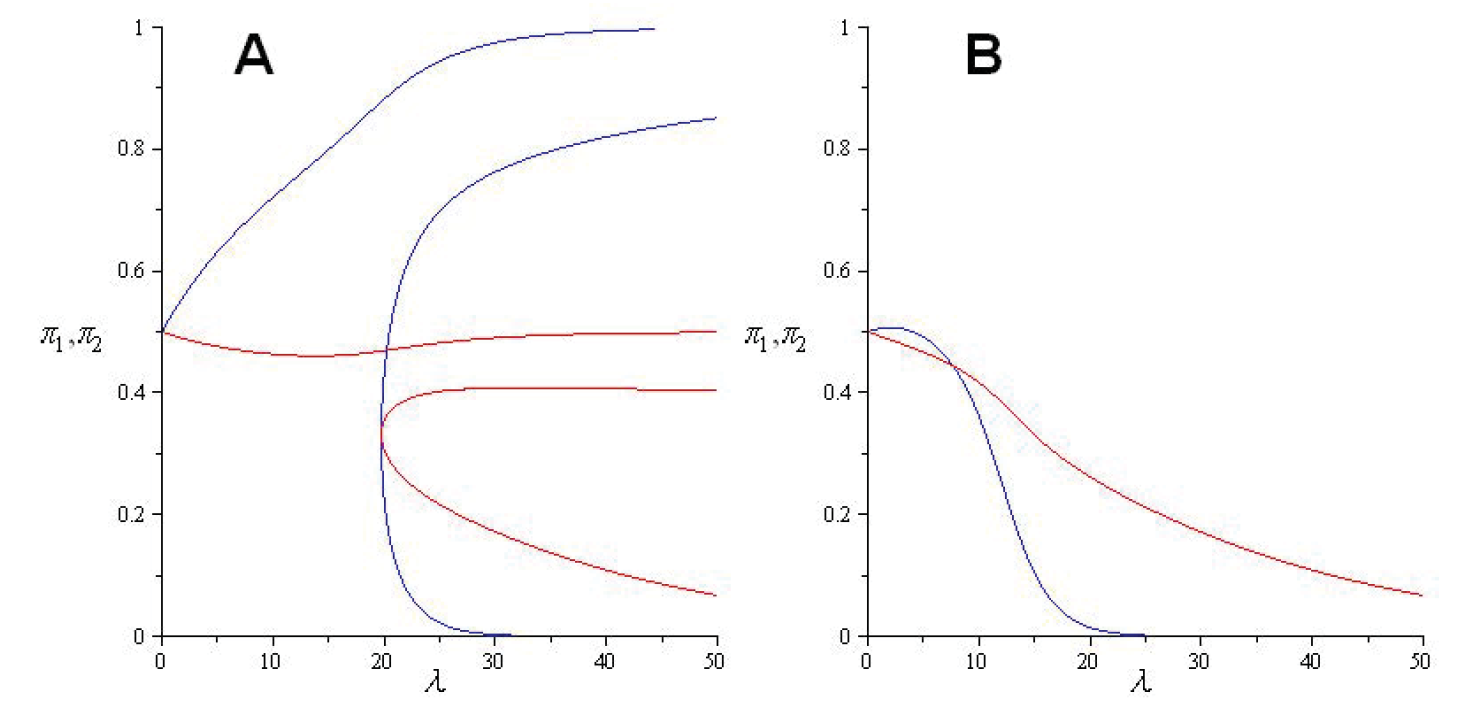

Supplement: Figure S1 — Graph of QRE correspondence. Parameters are taken as , in Figure S1A and in Figure S1B. Blue curve and red curve are and , respectively. In Figure S1A, since dominates 0.05, is the LLE. In Figure S1B, since 0.5 is dominated by 0.05, (0,0) is the LLE. (TIF) [file pone.0074540.s001.tif]

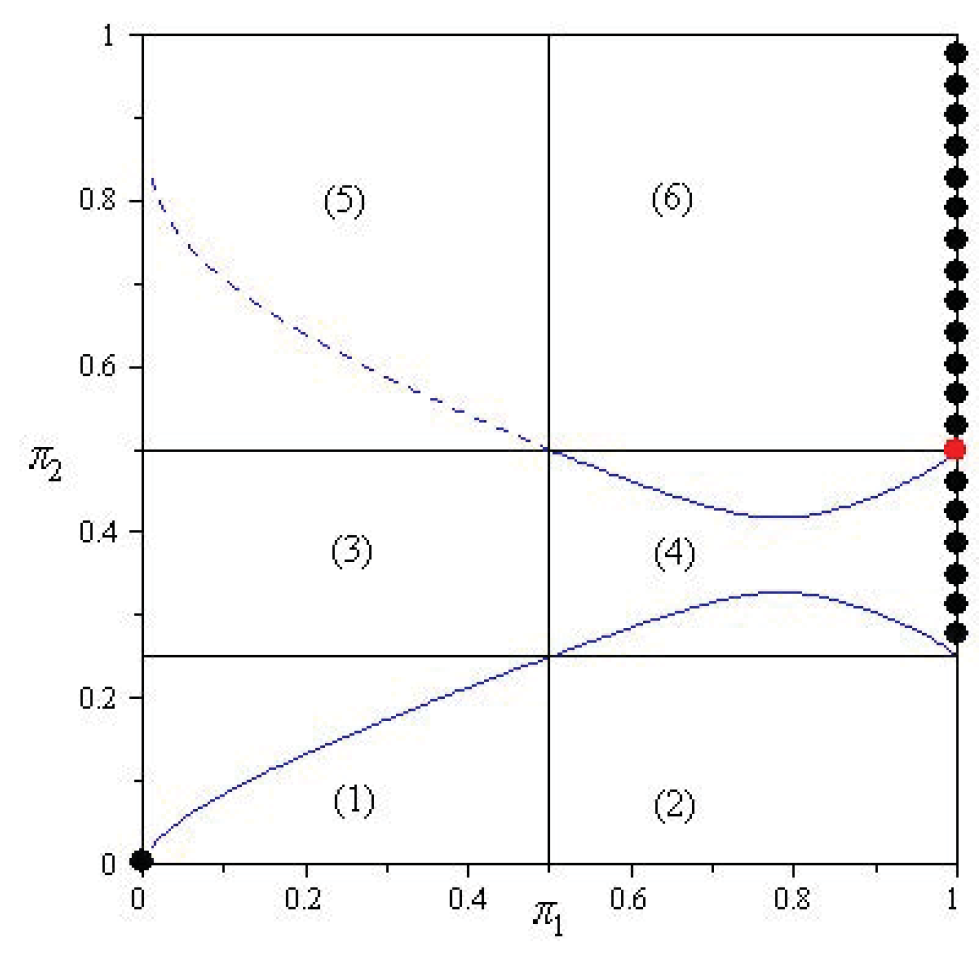

Supplement: Figure S2 — Graph of Eq.(S7). Parameters are taken as and , i.e., . on solid curves but on the dashed curve. Black points are Nash equilibria and the red point is the LLE. The graph of Eq.(S7) consists of two branches, where one passes through the Nash equilibrium and the other passes through the centroid . Since and are on the same branch, higher offer is the LLE. (TIF) [file pone.0074540.s002.tif]
