# Supplementary material for: Social Learning in the Ultimatum Game
Source: PLoS One. 2013 Sep 4;8(9):e74540. doi: 10.1371/journal.pone.0074540 (PMC3762740; doi:10.1371/journal.pone.0074540)
Supplement: Text S1 — Supporting Information for Social learning in the ultimatum game. (PDF) [file pone.0074540.s003.pdf]

Supporting Information for "Social learning in the ultimatum game"

Boyu Zhang[1] *

[1]School of Mathematical Sciences, Beijing Normal University, Beijing, P.R. China

Quantal response equilibrium

In a quantal response equilibrium (QRE), players are assumed boundedly rational and observe noisy evaluations of the strategies values. In a 2×2 bimatrix game, player i will choose the first strategy if and only if

$$u_{i1} + \varepsilon_{i1} > u_{i2} + \varepsilon_{i2}, \tag{S1}$$

where u_{ij} denotes the payoff of player i using strategy j and ε_{ij} denotes the observation noise on the strategy. In this case, best response function becomes probabilistic rather than deterministic. Suppose that player i's noise vector, $\varepsilon_i = (\varepsilon_{i1}, \varepsilon_{i2})$, is distributed according to a joint distribution with density function $p_i(\varepsilon_i)$, player i then adopts his first strategy with probability

$$\sigma_{i1}(u_{i1}, u_{i2}) = \int_{-\infty}^{\infty} \int_{-\infty}^{u_{i1}-u_{i2}+\varepsilon_{i1}} p_i(\varepsilon_i) d\varepsilon_{i2} d\varepsilon_{i1}, \tag{S2}$$

where $\sigma_{ij}(u_{i1}, u_{i2})$ is called the quantal response function.

*Corresponding Author: zhangboyu5507@gmail.com

The most common specification of QRE is the logit equilibrium. The logistic quantal response function is written as

$$\sigma_{i1}(u_{i1}, u_{i2}) = \frac{e^{\lambda u_{i1}}}{e^{\lambda u_{i1}} + e^{\lambda u_{i2}}} = \frac{1}{1 + e^{\lambda(u_{i2} - u_{i1})}}. \tag{S3}$$

This arises from Eq.(S2) if all the noises follow the extreme value distribution with cumulative distribution function $\exp(-\exp(-\lambda\varepsilon - \gamma))$, where γ is Euler's constant. Therefore, if each player uses a logistic quantal response function, the corresponding logit equilibria are solutions of

$$\begin{aligned} \pi_1 &= \frac{1}{1 + e^{\lambda(u_{12} - u_{11})}}, \\ \pi_2 &= \frac{1}{1 + e^{\lambda(u_{22} - u_{21})}}, \end{aligned} \tag{S4}$$

where $\pi_i = \pi_{i1}$ denotes the probability of player i using his first strategy. For the mini ultimatum game (1), Eq.(S4) is written as

$$\begin{aligned} \pi_1 &= \frac{1}{1 + e^{\lambda(1-l)(q-\pi_2)}}, \\ \pi_2 &= \frac{1}{1 + e^{\lambda l(1-\pi_1)}}, \end{aligned} \tag{S5}$$

where $q = \frac{h-l}{1-l}$.

Limiting logit equilibrium

Denote the set of QRE at noise level λ by G_λ. When $\lambda = 0$, Eq.(S5) has a unique solution, $G_0 = \{(\frac{1}{2}, \frac{1}{2})\}$, and when $\lambda \to +\infty$, QRE set consists of three Nash equilibria only, $G_{+\infty} = \{(0,0), (1, \frac{1}{2}), (1, q)\}$. Denote the graph of QRE correspondence by $G = \{(\lambda, \pi) | \lambda \geq 0, \pi \in G_\lambda\}$. We will show that for almost all mini ultimatum games, G includes a unique branch which starts for $\lambda = 0$ at $(\frac{1}{2}, \frac{1}{2})$ and converges to one of the Nash equilibria $(0, 0)$ and $(1, \frac{1}{2})$ as λ goes to infinity. This then defines a unique equilibrium selection by tracing the graph of QRE correspondence. (See Supporting Information Figure S1)

In order to show the structure of G, we look at the projection of Eq.(S5) on $\pi_1 - \pi_2$ plane. Notice that

$$\log \frac{1-\pi_1}{\pi_1} = \lambda(1-l)(q-\pi_2),$$
$$\log \frac{1-\pi_2}{\pi_2} = \lambda l(1-\pi_1), \tag{S6}$$

the projection is written as

$$(1-l)(q-\pi_2)\log \frac{1-\pi_2}{\pi_2} = l(1-\pi_1)\log \frac{1-\pi_1}{\pi_1}. \tag{S7}$$

From Eq.(S6), each solution of Eq.(S7) corresponds to a unique solution of Eq.(S5). Therefore, we turn to investigate the graph of Eq.(S7) instead of G.

Evidently, Eq.(S7) has two interior solutions $(\frac{1}{2}, \frac{1}{2})$ and $(\frac{1}{2}, q)$, and four boundary solutions $(0,0)$, $(0,1)$, $(1,q)$ and $(1, \frac{1}{2})$. (See Supporting Information Figure S2) The following theorem describes the graph of Eq.(S7).

Theorem S1

For almost all mini ultimatum games, the graph of Eq.(S7) consists of two (disjoint) branches, where one passes through the Nash equilibrium $(1,q)$ *and the other passes through the centroid* $(\frac{1}{2}, \frac{1}{2})$. *For the critical case, two branches intersect at a singular point.*

Proof:

Without loss of generality, suppose $0 < q < \frac{1}{2}$. We divide $\pi_1 - \pi_2$ plane to six regions by lines $x = \frac{1}{2}$, $y = q$ and $y = \frac{1}{2}$, and study the graph of Eq.(S7) in each region. Clearly, Eq.(S7) has no solution in regions (2), (3), and (6). Furthermore, $\lambda > 0$ in regions (1) and (4), and $\lambda < 0$ in region (5). (See Supporting Information Figure S2)

We next analyze the shape of solution curves in regions (1), (4) and (5) by the implicit function theorem. Define

$$S(\pi_1, \pi_2) = (1-l)(q-\pi_2)\log \frac{1-\pi_2}{\pi_2} - l(1-\pi_1)\log \frac{1-\pi_1}{\pi_1}. \tag{S8}$$

The derivatives of $S(\pi_1,\pi_2)$ satisfy

$$\begin{aligned}
S_1 &= l\log\frac{1-\pi_1}{\pi_1}+\frac{l}{\pi_1},\\
S_{11} &= -\frac{l}{\pi_1(1-\pi_1)}-\frac{l}{\pi_1^2},\\
S_2 &= -(1-l)\log\frac{1-\pi_2}{\pi_2}-\frac{(1-l)(q-\pi_2)}{(1-\pi_2)\pi_2},\\
S_{22} &= \frac{1-l}{(1-\pi_2)\pi_2}+\frac{(1-l)(1-q)(1-2\pi_2)}{((1-\pi_2)\pi_2)^2}+\frac{1-l}{(1-\pi_2)\pi_2},
\end{aligned} \tag{S9}$$

where $S_1>0$ if $\pi_1<\frac{1}{2}$, $S_{11}\le 0$, $S_2<0$ if $\pi_2<q$, $S_2>0$ if $\pi_2>\frac{1}{2}$ and $S_{22}\ge 0$ if $q<\pi_2<\frac{1}{2}$.

Hence, in region (1), $S(\pi_1,\pi_2)=0$ is an increasing curve from $(0,0)$ to $(\frac{1}{2},q)$; in region (5), $S(\pi_1,\pi_2)=0$ is a decreasing curve from $(0,1)$ to $(\frac{1}{2},\frac{1}{2})$. On the other hand, in region (4), we have $S_{11}\le 0$ and $S_{22}\ge 0$, i.e., S is a concave function of π_1 and a convex function of π_2. This implies that for given $\hat{\pi}_2$, $S(\pi_1,\pi_2)=0$ has (a) two solutions $(\pi_1',\hat{\pi}_2)$ and $(\pi_1'',\hat{\pi}_2)$ if $S(\pi_1^*,\hat{\pi}_2)>0$, (b) one solution $(\pi_1^*,\hat{\pi}_2)$ if $S(\pi_1^*,\hat{\pi}_2)=0$, (c) no solution if $S(\pi_1^*,\hat{\pi}_2)<0$, where $\pi_1'<\pi_1^*<\pi_1''$ and π_1^* is the solution of $S_1(\pi_1^*)=0$. Similarly, for given $\hat{\pi}_1$, $S(\pi_1,\pi_2)=0$ has (d) two solutions $(\hat{\pi}_1,\pi_2')$ and $(\hat{\pi}_1,\pi_2'')$ if $S(\hat{\pi}_1,\pi_2^*)>0$, (b) one solution $(\hat{\pi}_1,\pi_2^*)$ if $S(\hat{\pi}_1,\pi_2^*)=0$, (c) no solution if $S(\hat{\pi}_1,\pi_2^*)<0$, where $\pi_2'<\pi_2^*<\pi_2''$ and π_2^* is the solution of $S_2(\pi_2^*)=0$. Thus, the graph of $S(\pi_1,\pi_2)=0$ in region (5) consists of two disjoint curves that are separated by line $\pi_1=\pi_1^*$ if $S(\pi_1^*,\pi_2^*)>0$ (from (a) and (f)) and by line $\pi_2=\pi_2^*$ if $S(\pi_1^*,\pi_2^*)<0$ (from (c) and (d)).

In sum, if $S(\pi_1^*,\pi_2^*)\neq 0$, the graph of $S(\pi_1,\pi_2)=0$ consists of two disjoint branches, where the Nash equilibrium $(1,q)$ and the centroid $(\frac{1}{2},\frac{1}{2})$ are always on different branches; if $S(\pi_1^*,\pi_2^*)=0$, two branches intersect at the point (π_1^*,π_2^*). $\square$

From Theorem S1, the LLE is $(0,0)$ (or $(1,\frac{1}{2})$) if and only if $(0,0)$ (or $(1,\frac{1}{2})$) and $(\frac{1}{2},\frac{1}{2})$ are on the same branch. On the other hand, $(1,q)$ can not be selected for almost all mini ultimatum games since there is no path from $(\frac{1}{2},\frac{1}{2})$ to it.

In the critical case, two branches intersect at a singular point (π_1^*,π_2^*) and tracing the branch of QRE correspondence beginning at the centroid could reach all three Nash

equilibria. In this case, we have $S(\pi_1^*, \pi_2^*) = 0$, $S_1(\pi_1^*) = 0$ and $S_2(\pi_2^*) = 0$, i.e.

$$
\begin{aligned}
&(1-l)(q-\pi_2^*)\log\frac{1-\pi_2^*}{\pi_2^*} - l(1-\pi_1^*)\log\frac{1-\pi_1^*}{\pi_1^*} = 0,\\
&\log\frac{1-\pi_1^*}{\pi_1^*} + \frac{1}{\pi_1^*} = 0,\\
&\log\frac{1-\pi_2^*}{\pi_2^*} + \frac{q-\pi_2^*}{(1-\pi_2^*)\pi_2^*} = 0.
\end{aligned} \tag{S10}
$$

Define $f(\pi_i) = \pi_i - \pi_i(1-\pi_i)\log\frac{1-\pi_i}{\pi_i}$. The second and the third equations of Eq.(S10) are then written as $f(\pi_1^*) = 1$ and $f(\pi_2^*) = q$. It is easy to check that $f(\pi_i)$ is increasing in π_i and $f(\frac{1}{2}) = \frac{1}{2}$. Thus, π_1^* and π_2^* in the first equation of Eq.(S10) could be replaced by $f^{-1}(1)$ and $f^{-1}(q)$, respectively, i.e.,

$$(1-l)(q-f^{-1}(q))\log\frac{1-f^{-1}(q)}{f^{-1}(q)} = l(1-f^{-1}(1))\log\frac{1-f^{-1}(1)}{f^{-1}(1)}. \tag{S11}$$

For convenience, define $F(q) = \sqrt{(q-f^{-1}(q))\log\frac{1-f^{-1}(q)}{f^{-1}(q)}}$, Eq.(S11) is then simplified as

$$F(q) = -\sqrt{\frac{l}{1-l}}F(1). \tag{S12}$$

In order to get an explicit expression of Eq.(S12), we substitute $F(q)$ and $F(1)$ in Eq.(S12) by their Taylor expansion at $\frac{1}{2}$

$$F(q) = (q-\frac{1}{2}) + \frac{1}{6}(q-\frac{1}{2})^3 + \frac{17}{120}(q-\frac{1}{2})^5 + o(q-\frac{1}{2})^7. \tag{S13}$$

Finally, we obtain

$$q = \frac{1}{2}(1-\sqrt{c}) + \frac{1}{2^3}\frac{\sqrt{c}}{6}(c-1) + \frac{1}{2^5}\frac{\sqrt{c}}{120}(7c^2+10c-17) + o(\frac{1}{2^7}), \tag{S14}$$

where $c = \frac{l}{1-l}$. One can see that high order terms in Eq.(S14) are very small. In fact, if the lower offer is less than 0.5, i.e., $c < 1$, the absolute value of the sum of all high order terms is less than 0.01. This implies that Eq.(S14) could be well approximated by its linearization

$$q = \frac{1}{2}(1-\sqrt{\frac{l}{1-l}}). \tag{S15}$$

From the proof of Theorem S1, the LLE is $(1, \frac{1}{2})$ if and only if $q < \frac{1}{2}(1-\sqrt{\frac{l}{1-l}})$, i.e.,

$$2h < l + 1 - \sqrt{l(1-l)}. \tag{S16}$$
